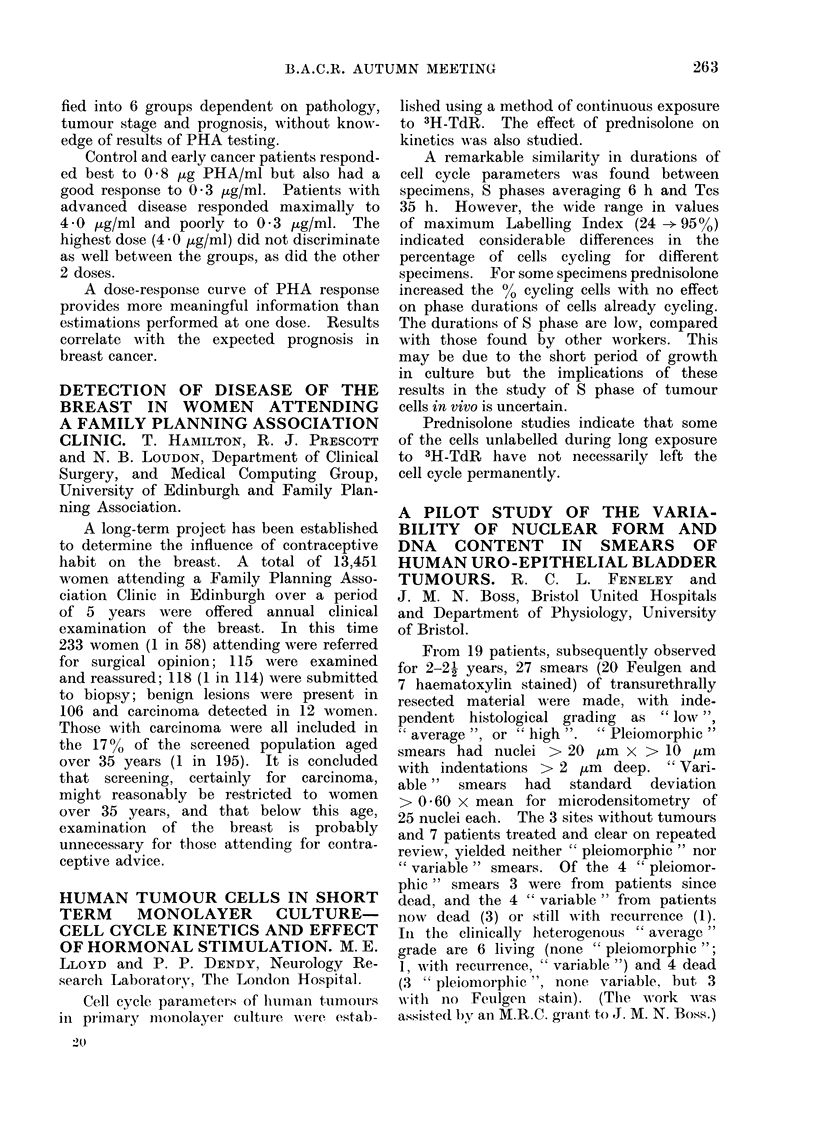# Proceedings: Detection of disease of the breast in women attending a family planning association clinic.

**DOI:** 10.1038/bjc.1975.46

**Published:** 1975-02

**Authors:** T. Hamilton, R. J. Prescott, N. B. Loudon


**DETECTION OF DISEASE OF THE BREAST IN WOMEN ATTENDING A FAMILY PLANNING ASSOCIATION CLINIC.** T. HAMILTON, R. J. PRESCOTT and N. B. LOUDON, Department of Clinical Surgery, and Medical Computing Group, University of Edinburgh and Family Planning Association.

A long-term project has been established to determine the influence of contraceptive habit on the breast. A total of 13,451 women attending a Family Planning Association Clinic in Edinburgh over a period of 5 years were offered annual clinical examination of the breast. In this time 233 women (1 in 58) attending were referred for surgical opinion; 115 were examined and reassured; 118 (1 in 114) were submitted to biopsy; benign lesions were present in 106 and carcinoma detected in 12 women. Those with carcinoma were all included in the 17% of the screened population aged over 35 years (1 in 195). It is concluded that screening, certainly for carcinoma, might reasonably be restricted to women over 35 years, and that below this age, examination of the breast is probably unnecessary for those attending for contraceptive advice.